# Vaccination against *Clostridium perfringens* type C enteritis in pigs: a field study using an adapted vaccination scheme

**DOI:** 10.1186/s40813-019-0127-8

**Published:** 2019-08-15

**Authors:** Olivia K. Richard, Alexander Grahofer, Heiko Nathues, Horst Posthaus

**Affiliations:** 1 Institute of Animal Pathology, Department of Infectious Diseases and Pathobiology, Vetsuisse Faculty, University of Bern, Längassstrasse 122, 3012 Bern, Switzerland; 2 Clinic for Swine, Department of Clinical Veterinary Science, Vetsuisse Faculty, University of Bern, 3012 Bern, Switzerland; 3 Present address: Department of Environmental Systems Science, ETH Zürich, 8092 Zürich, Switzerland

**Keywords:** *Clostridium perfringens* type C, Beta-toxin, Neutralizing antibodies, Necrotizing enteritis, Porcine, Vaccination

## Abstract

**Background:**

*Clostridium perfringens* type C induced necrotizing enteritis (NE) causes high mortality in newborn piglets. Immunization programs employing commercially available vaccines are used to prevent disease. Sows are vaccinated during every gestation period and piglets take up antibodies from the colostrum. Antibodies against the major clostridial toxin beta-toxin (CPB) are considered essential for protective immunity. Because the pathogen can persist for several years on farms, continuous vaccination is essential to protect pig herds from the re-occurrence of NE.

**Results:**

In two field trials using commercially available vaccines we monitored neutralizing anti-CPB antibodies in pigs after vaccination. The first trial compared antibody titers in primiparous (gilts) and multiparous sows and their piglets after vaccination. A proportion of gilts and their piglets’ showed no or low antibody titers. All multiparous sows developed significantly higher serum and colostrum antibody titers after a booster vaccination shortly before their next farrowing. These colostral antibody titer highly correlated with the serum antibody titer of their piglets after consumption of colostrum. In a second field trial, we adapted the vaccination schemes using 3 instead of 2 initial vaccinations before the first farrowing of gilts. This significantly increased serum and colostrum antibody titers in gilts and serum antibody titers in piglets.

**Conclusion:**

We demonstrate that despite following recommended vaccination protocols, a proportion of gilts might not sufficiently seroconvert to provide efficient passive immunity to their offsprings. A simple adaptation of the vaccination scheme can however improve passive protection of piglets from NE.

## Background

*Clostridium perfringens* type C causes necrotizing enteritis (NE) in neonatal pigs and can lead to significant economic losses on pig breeding farms [1]. Protection against NE is achieved by vaccination of sows with commercially available type C toxoid vaccines [2, 3]. Because *C. perfringens* type C can persist on farms over long periods, long-term vaccination should remain despite the eradication of the disease from once affected herds [4, 5]. If sufficient protective antibody levels in the sow colostrum are achieved, piglets are passively protected by the uptake of antibodies via the colostrum and milk of the sows [2]. The exact amount and isotype of antibodies, which provide full protection to piglets under field conditions, are however not known. *C. perfringens* beta-toxin (CPB), has been shown to be the essential virulence factor for the pathogenesis of NE [6–8]. It is likely, that antibodies neutralizing its effect play a major role in protecting piglets from NE. The results of few studies on in pigs [3, 9–13] and laboratory animal challenge models [2, 14] suggest that anti-CPB antibodies are a useful indicator of immunity against *C. perfringens* type C enteritis. This is supported by epidemiological data showing that vaccination largely reduces the incidence of NE on pig breeding farms [2, 15, 16].

The applied vaccination scheme can influence the levels of antibodies in sow colostrum and milk, and thus protection against disease [12, 13]. Currently it is recommended to vaccinate primiparous sows (gilts) twice after insemination and before their first farrowing followed by one booster vaccination prior to every subsequent farrowing [2, 3, 13, 15]. Nevertheless, we and others have experienced that NE occasionally still re-occurs in immunized herds [15, 17]. Failure of piglets to receive adequate amounts of protective antibodies via colostrum and milk, trypsin secretion deficiencies in piglets, and colostral trypsin inhibitors are factors discussed to contribute to such outbreaks [1].

In our current study, we evaluated the development of neutralizing anti-CPB antibodies in serum and colostrum of vaccinated gilts and multiparous sows under field conditions. In addition, we investigated serum neutralizing anti-CPB antibody levels in piglets as an indicator for the transfer of antibodies to the offspring of vaccinated sows. We performed investigations on three farms, which vaccinated against *C. perfringens* type C, and one farm that served as negative control. According to the results of this first investigation, we subsequently evaluated an adapted vaccination scheme using two initial vaccine injections as basic immunization before insemination and one booster immunization before the first farrowing.

## Results

### First field investigation

The aim of our first field trial was to evaluate neutralizing anti-CPB antibody titers as a measurement for protection against NE under practical conditions on selected Swiss breeding farms. We additionally compared antibody titers of gilts with those of multiparous sows. The study was conducted to evaluate regular vaccination practices used on these farms and rather than to compare different vaccines, thus we grouped all vaccinated sows independent of their origin (farm) and the vaccine used anticipating that the source of vaccine has no effect.

**Fig. 1:**
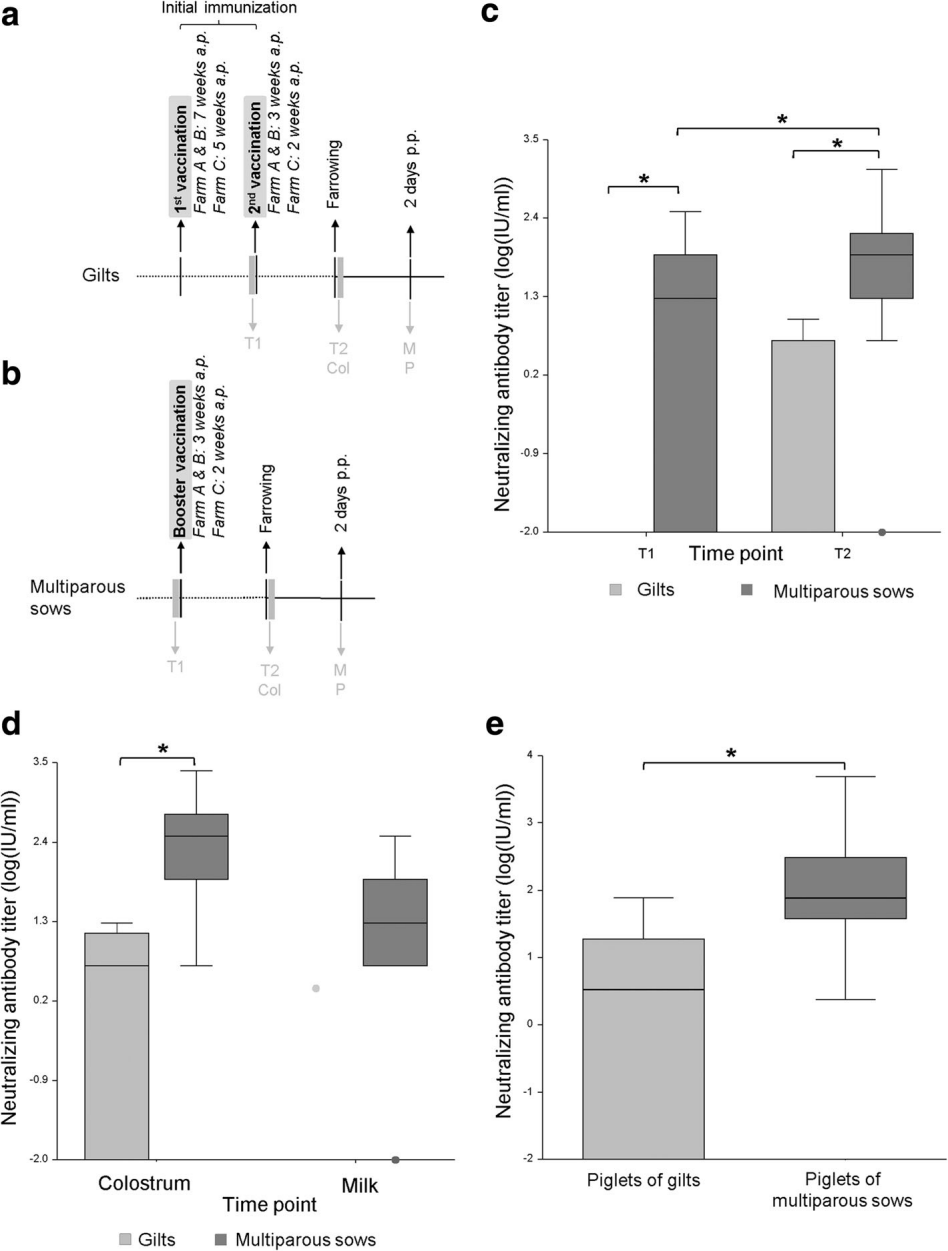
Comparison of neutralizing anti-CPB antibody titers in gilts and multiparous sows and their piglets. **a** & **b**: Immunization and sampling schedule for field trial. **c**, **d** & **e**: Neutralizing anti-CPB antibodies in gilts, multiparous sows and piglets on farms vaccinating against *C. perfringens* type C. **a**. Timeline of sample collection in the first field trial for gilts: T1: before the second vaccination, T2 and Col: in the first 24 h *postpartum* (*p.p.)*, M, P: 2 days *p.p.*; T: blood sample of the sow, Col: colostrum, M: milk, P: blood samples of piglets. **b**. Timeline of sample collection in the first field trial for multiparous sows: T1: before the booster vaccination, T2 and Col: in the first 24 h *p.p.*, M, P: 2 days *p.p.*; T: blood sample of the sow, Col: colostrum, M: milk, P: blood samples of piglets. **c**. Neutralizing anti-CPB antibody titers (logarithmic scale) in serum of gilts and multiparous sows. Serum from gilts was taken before (T1) and after the second vaccination (T2), serum of multiparous sows before (T1) and after the booster vaccination (T2) of subsequent farrowings. Box plots depicting median, quartiles and range. Antibody titers in gilts at T2 ranged from 0 to 9.54 IU/ml with a median of 0 IU/ml, whereas titers in sows were significantly higher (*p* < 0.01 for all time points). ANOVA, Tukey-Kramer Multiple-Comparison Test. **d**. Neutralizing anti-CPB antibody titers in colostrum (Col) and milk (M) samples of gilts and multiparous sows. Colostral and milk antibody titers of multiparous sows were significantly higher than antibody titers of gilts (*p < 0.01 for all time points and groups). ANOVA, Tukey-Kramer Multiple-Comparison Test. **e**. Neutralizing anti-CPB antibody titers in serum of piglets from gilts (0) and multiparous sows (1). Antibody titers from piglets of multiparous sows were significantly higher than antibody titers from piglets of gilts (*p < 0.01). ANOVA, Tukey-Kramer Multiple-Comparison Test

On farms A-C, which continuously vaccinated against *C. perfringens* type C enteritis (vaccination scheme Fig. 1a, b), 4 out of the total 9 gilts, gained no neutralizing anti-CPB antibody titers in serum or colostrum samples. In 5 of 9 gilts serum antibody titers ranging from 4.77–9.54 IU (Fig. 1c) and colostrum antibody titers ranging from 4.77–19.08 (Fig. 1d) were detected. Overall, this resulted in median neutralizing anti-CPB antibody titers of 0 IU/ml in serum and 4.77 IU/ml in colostrum of gilts. Except for one sample (2.38 IU/ml), all milk samples of gilts, taken two to three days after the farrowing, were negative for neutralizing anti-CPB antibodies (Fig. 1d). In 6 of the piglets from gilts, no serum antibody titers could be detected. The remaining 12 piglets showed antibody titers ranging from 2.385–76.32 IU (Fig. 1e). The median of the antibody titers of piglets of gilts was 3.57 IU/ml. Multiparous sows showed significantly higher serum antibody titers after the booster vaccination when compared to titers before the booster vaccination and to those in gilts (Fig. 1c), ranging from 4.77–1221.12 IU with a median of 76.32 IU/ml. Colostral antibody titers were detectable in every multiparous sow and were also significantly higher than in gilts (range: 4.77–2442.24 IU/ml; median: 305.28 IU/ml; Fig. 1d). In addition, neutralizing anti-CPB antibodies were detectable in 32 of 36 milk samples of multiparous sows, taken two to three days after farrowing (range: 4.77–305.8 IU/ml; median 19.08 IU/ml; Fig. 1d).

Antibody titers in serum of piglets from multiparous sows ranged from 2.385–4884.48 IU/ml (median 76.32 IU/ml; Fig. 1e) and were significantly higher when compared to piglets from gilts (*p* < 0.01, Fig. 1e). The colostral antibody titers of multiparous sows strongly correlated with the antibody titers of their piglets (Correlation coefficient: 0.79). Their association can be described with the following regression model: Log (Y + 0.01) = 0.5614 + Log (X + 0.01)*0.5904, where Y is the antibody titer of the serum and X the antibody titer of the colostrum (*p*-value < 0.01, R2 = 0.5608).

As a control, we included a non-vaccinating farm which did not experience NE over more than 3 years prior to the start of the study. In all serum and colostrum samples from this farm, we were unable to detect neutralizing antibodies against CPB.

### Second field investigation using an adapted vaccination scheme

**Fig. 2:**
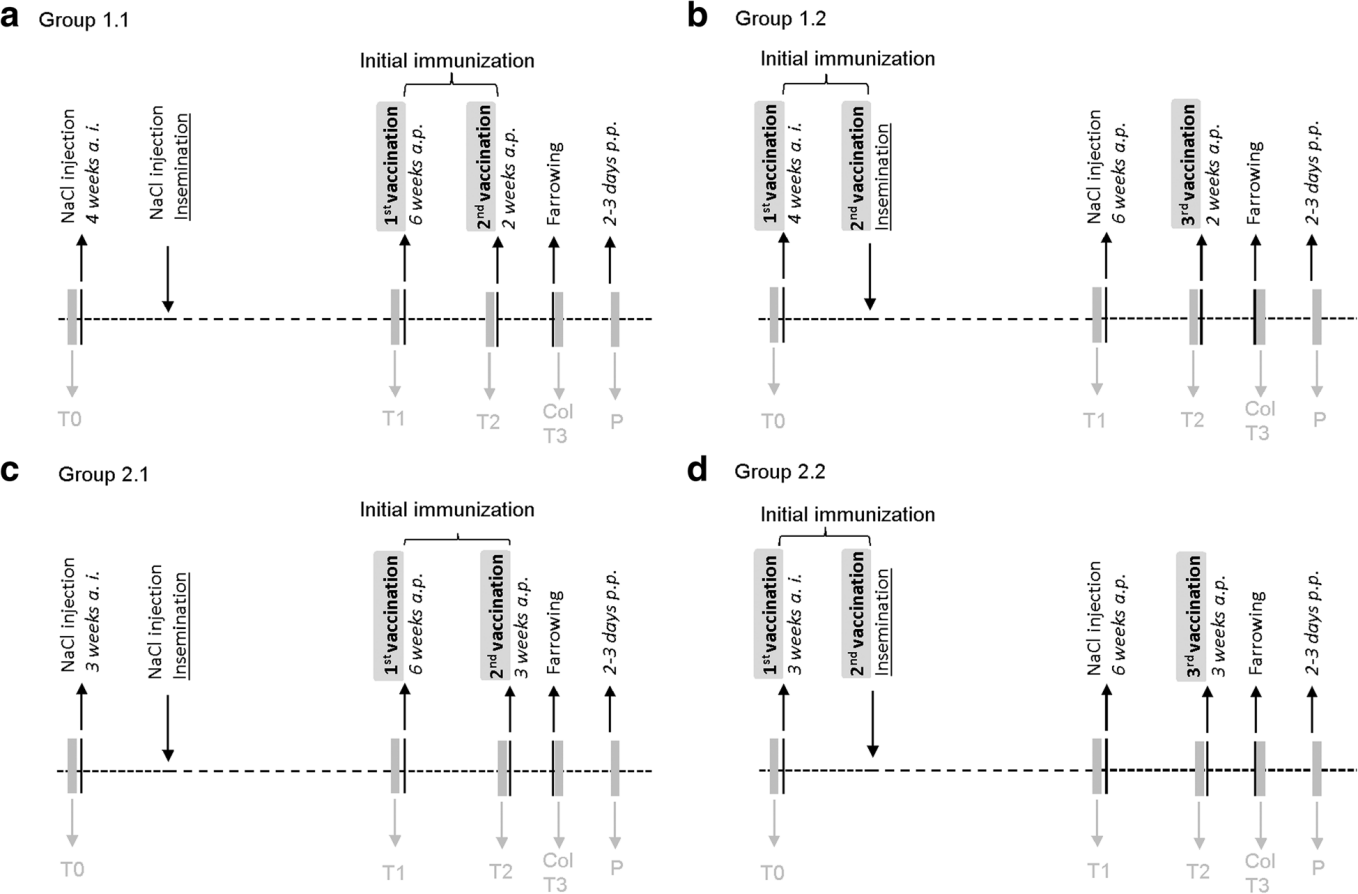
Immunization and sampling schedule for second field trial. **a**. Standard vaccination scheme (2 applications), Porcilis® ColiClos ad us. vet. (Group 1.1). **b**. Adapted vaccination scheme (3 applications), Porcilis® ColiClos ad us. vet. (Group 1.2). **c**. Standard vaccination scheme (2 applications), Suisen ad us. vet. (Group 2.1). **d**. Adapted vaccination scheme (3 applications), Suisen ad us. vet. (Group 2.2). Timeline of sample collection in the vaccination trial; T0: before vaccination, 3 or 4 weeks ante insemination (a. i.), T1: 5 or 6 weeks *ante-partum*, T2: 2 or 3 weeks *ante-partum*, T3: at farrowing, P: 2 to 3 days *postpartum (p.p.)*; T: blood sample of the sow, Col: colostrum, P: blood samples of piglets, NaCl injections were performed to achieve even numbers of injections between all groups

In the first part of our study, we detected relatively low or even no titers of neutralizing anti-CPB antibodies in gilts and their piglets despite following the manufacturers recommended vaccination scheme. We next wanted to test whether an adapted vaccination scheme, using three instead of two initial vaccine injections for gilts leads to increased neutralizing anti-CPB antibody titers (Fig. 2). For this purpose, we purchased and used the two *C. perfringens* type C vaccines, which were licensed and commercially available at that time in Switzerland. Both contain inactivated *C. perfringens* type C culture supernatants. Gilts in the control groups (group 1.1 and 2.1) were vaccinated following the manufacturer recommendation against *C. perfringens* type C, which included two injections of the vaccine *ante-partum* (a.p.). Gilts in the groups with the adapted vaccination scheme (group 1.2 and 2.2) were vaccinated two times prior to insemination and received an additional booster vaccination prior to farrowing.

**Fig. 3:**
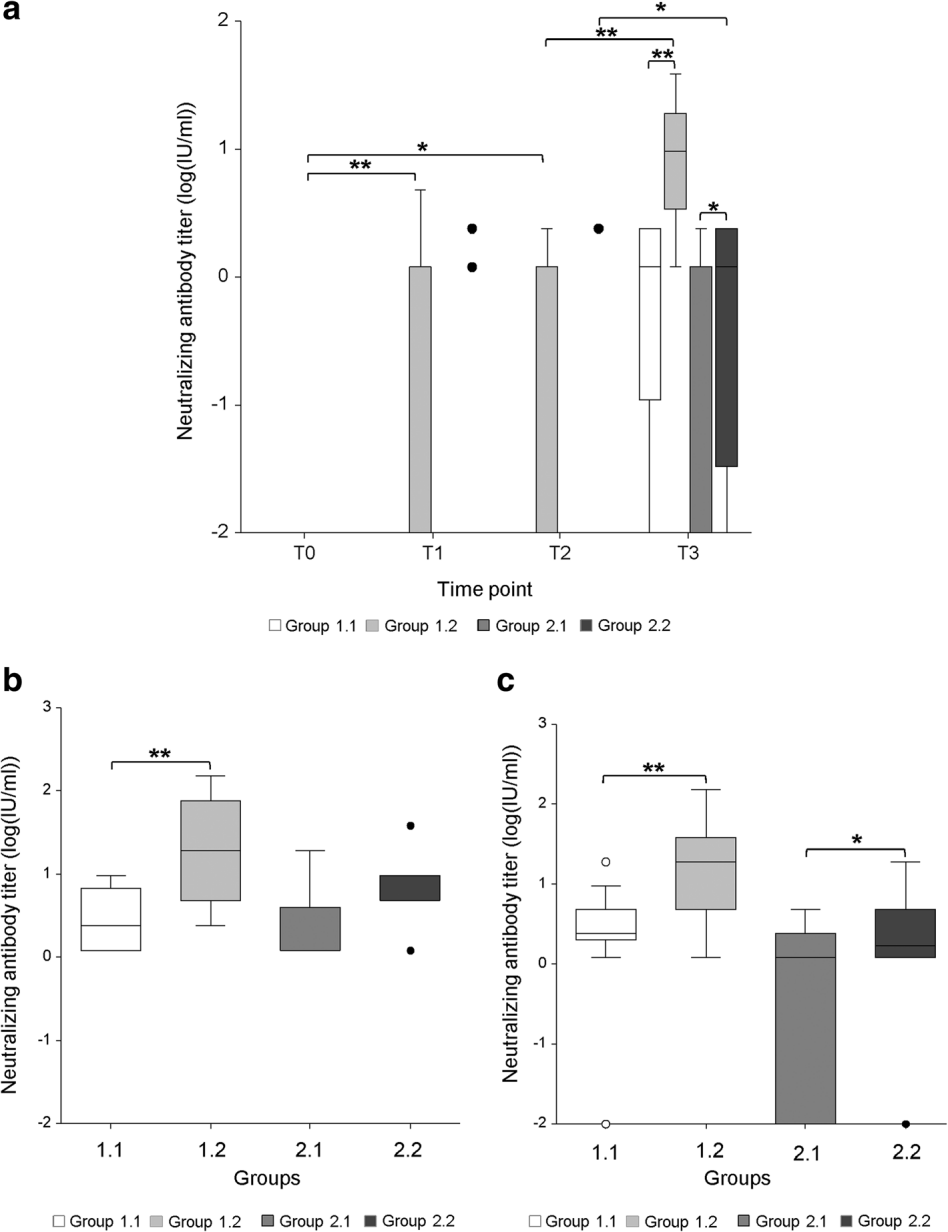
Neutralizing anti-CPB antibody titers in gilts and their piglets using an adapted vaccination scheme. Group 1.1: standard vaccination scheme vaccine 1; Group 1.2: adapted vaccination scheme vaccine 1; Group 2.1: standard vaccination scheme vaccine 2; Group 2.2: standard vaccination scheme vaccine 2. **a**. Antibody titers in serum samples of gilts. Serum antibody titers of gilts at time of farrowing (T3) from groups with adapted vaccination scheme (1.2 and 2.2) were significantly higher compared to the standard scheme using the identical vaccine (1.1 and 2.1). (* = *p* < 0.05; ** = *p* < 0.01). ANOVA, Tukey-Kramer Multiple-Comparison Test. **b**. Antibody titers in colostrum samples of gilts. Colostral antibody titers of gilts from group 1.2 with the adapted vaccination scheme were significantly higher compared to the standard scheme using the identical vaccine (1.1) (** = *p* < 0.01). The elevation of antibody titers in group 2.2 was statistically non-significant compared to group 2.1. There was no statistical significance between group 2.1 and 2. ANOVA, Tukey-Kramer Multiple-Comparison Test. **c**. Antibody titers in serum samples of piglets. Serum antibody titers of piglets from groups with the adapted vaccination scheme (1.2 and 2.2) were significantly higher compared to the standard scheme using the identical vaccine (1.1 and 2.1). (* = *p* < 0.05; ** = *p* < 0.01) ANOVA, Tukey-Kramer Multiple-Comparison Test

Prior to vaccination, no neutralizing anti-CPB antibodies were detected in any of the four groups of gilts. In Group 1.1 (Fig. 2a, standard vaccination scheme, vaccine 1) no serum antibodies were detected before and after the first vaccination (Fig. 3a). After the second vaccination, at time of farrowing, 3 out of 13 sows did not gain detectable serum neutralizing anti-CPB antibody titers, the other 10 sows showed antibody titers ranging from 1.19–2.38 IU/ml. The overall median antibody titer in this group was 1.19 IU/ml. (Fig. 3a). All sows had detectable colostrum antibody titers ranging from 1.19–9.54 IU/ml, with a median of 2.39 IU/ml. Three out of 26 piglets did not gain detectable serum antibody titers, the other 23 gained serum antibodies ranging from 1.19–19.08 IU/ml. The overall median antibody titer in this group of piglets was 2.39 IU/ml (Fig. 3c).

In Group 1.2 (Fig. 2b, adapted vaccination scheme, vaccine 1), 4 and 5 months after the basic immunization 5 of 12 gilts did not gain detectable serum antibody titers, whereas 7 gilts gained antibody titers ranging from 1.19–4.77 IU/ml. This resulted in a median serum antibody titer of 1.19 IU/ml (Fig. 3a). At time of farrowing after the booster vaccination, all gilts had detectable serum and colostrum antibody titers (range serum: 1.19–38.16 IU/ml, median: 9.54 IU/ml; range colostrum: 2.38–152.64 IU/ml, median colostrum: 19.08 IU/ml) (Fig. 3a, b). The median antibody titer in the colostrum of gilts was thus approximately 8x higher compared to group 1.1 (*p*-value: < 0.01). All piglets gained detectable serum antibody titers (range: 1.19–152.64 IU/ml, median: 19.08 IU/ml). Compared to the piglets from group 1.1 the median antibody titer was 7.9x times higher in the group with the adapted vaccination scheme (Group 1.2; *p*-value: < 0.01) (Fig. 3c).

In Group 2.1 (Fig. 2c, standard vaccination scheme, vaccine 2) no serum antibodies were detected in gilts before and after the first vaccination. At time of farrowing 9 out of 16 sows did not gain a detectable serum antibody titer, the other 7 sows did gain antibody titers ranging from 1.19–2.38 IU/ml (Fig. 3a). The overall median antibody titer was 0 IU/ml. All sows gained detectable colostrum antibody titers (range: 1.19–19.08 IU/ml, median: 1.19 IU/ml) (Fig. 3b). Nine out of 31 piglets did not gain any detectable serum antibody titer, the other 22 gained serum antibody titers ranging from 1.19–4.77, resulting in a median of 1.19 IU/ml (Fig. 3c).

In group 2.2 (Fig. 2d, adapted vaccination scheme, vaccine 2) two of 12 sows gained antibody titers measured 4 months after the basic immunization (1.19 IU/ml, 2.38 IU/ml respectively). At time of farrowing, after the booster vaccination, 3 of 12 sows did not gain a detectable serum antibody titer, the other 9 did gain a serum antibody titer ranging from 1.19–2.38 IU/ml (Fig. 3a). The overall median antibody titer value was 1.19 IU/ml. All sows gained detectable colostral antibody titers (range1.19–38.16 IU/ml, median: 4.77 IU/ml) (Fig. 3b). Compared to group 2.1 the median colostrum antibody titer was 4x times higher in group 2.2, but this result was statistically insignificant (*p*-value: > 0.05). One piglet of 26 did not gain a detectable serum antibody titer, the other 25 piglets gained antibody titers ranging from 1.19–19.08 IU/ml, resulting in a median antibody titer of 1.79 IU/ml (Fig. 3c). Thus, serum antibody titers of group 2.2 were approximately 1.5 x higher than those of group 2.1 and this difference was statistically significant (*p*-value: < 0.05).

## Discussion

The first aim of our study was to evaluate the levels of neutralizing anti-CPB antibody titers induced by routine vaccination against *C. perfringens* type C enteritis in sows and their piglets under practical condition in Swiss pig breeding farms. As CPB is the essential toxin secreted by *C. perfringens* type C strains [6], such antibody titers are a useful indicator for passive immunity against NE, as they correlate with protection against the effects of *C. perfringens* type C supernatants in lethal challenge models in pigs [9]. Our results clearly show that different commercially available vaccines effectively induce development of antibodies capable of neutralizing CPB in vaccinated sows. We could clearly demonstrate that booster vaccinations before every farrowing leads to the development of high colostrum and even milk antibody titers in multiparous sows and that these antibodies were transferred to their offspring. As our study was primarily designed to determine CPB neutralizing activity we did not determine the Ig class of these antibodies. However, taking studies about IgA and IgG in colostrum and milk of sows into account [18, 19], it can be anticipated that colostrum derived antibodies belonging to the class of IgG are crucially important for the development of immunity against NE in piglets. It seems likely that also lactogenic transferred IgA adds to a passive mucosal immunity against CPB and potentially other clostridial toxins, but the contribution of colostral versus lactogenic immunity would still have to be investigated in more detail using different experimental setups. The absence of neutralizing antibodies in milk from gilts observed in our first field trial however is a further indication for a lower passive immunity and thus elevated susceptibility of piglets from gilts towards development of NE.

Studies evaluating the minimal antibody titer, which confers protective immunity in piglets against NE under field condition, are lacking, thus it is not possible to scientifically demonstrate protection against NE in our study. According to Hogh [3] titers of 10 IU/ml of beta-antitoxin in colostral whey should be considered as sufficient for protecting piglets against NE. These titers were however determined differently from our method and do not necessarily reflect antibodies against CPB only. Nevertheless, neutralizing anti-CPB antibody titers of 10 IU/ml and higher were readily reached in multiparous sows after booster immunizations, suggesting that the applied vaccination scheme was sufficient to induce protective immunity in their piglets.

Interestingly, similar to our previous results using samples of a vaccine efficacy trial [9], also under field conditions, gilts frequently developed low colostrum antibody titers, which resulted in much lower antibody titers in their piglets compared to multiparous sows, which were continuously boostered during every gestation. Individual piglets even showed no detectable neutralizing anti-CPB serum antibody titers. Very early studies in the 60ies and 70ies showed that oral and also s.c. administration of beta-antitoxin leads to detectable titers of antibodies in piglet serum and actually leads to partial protection of piglets against NE [3, 16]. Application of antiserum to piglets was even suggested as an alternative option to vaccination of sows to reduce the incidence of NE by these authors at that time. Currently it is still unknown, which antibodies transferred from the colostrum to the piglets prevent NE and how exactly such antibodies interfere with the pathogenesis of NE. The pathogenesis of NE involves an initial overgrowth of *C. perfringens* type C, secretion of multiple toxins including targeted effects of CPB, mainly on endothelial cells in the small intestinal mucosa [1, 7, 20] (Posthaus et al. submitted to JVDI). It is likely that IgA antibodies against several clostridial virulence factors, amongst them CPB, taken up with colostrum and milk have a direct protective effect in the intestinal lumen. Due to the high susceptibility of endothelial cells to CPB, circulating neutralizing IgG antibodies against CPB might also be important in preventing the development of the necro-hemorrhagic lesions observed in NE. Our results, which focus on the levels of anti-CPB antibodies that neutralize the toxic effects of this essential virulence factor of *C. perfringens* type C, thus suggest that piglets from gilts are at higher risk to develop disease once they ingest *C. perfringens* type C shortly after birth. This might explain our observation that in outbreaks of NE on pig breeding farms, which vaccinate against *C. perfringens* type C, mainly piglets from gilts, succumb to disease (Nathues, personal observation).

In the second part of our study, we demonstrated that an adaptation of the vaccination scheme using three instead of two vaccine injections for the basic immunization of gilts leads to a marked and significant increase in antibody levels in colostrum and piglets, respectively. This result was independent of the vaccine used. Thus in cases where needed, this very simple adaptation can be used to increase the level of immunity in the most vulnerable subgroup of the pig population. We detected differences in antibody titers induced by two different commercially available vaccines. However, it was beyond the scope of our study to compare different products, which would require much larger cohorts of animals and groups to be tested.

It should be noted that under practical conditions it seems to be generally sufficient to use a two-injection basic immunization protocol for gilts. This has to be followed by strict enforcement of one booster vaccination during every subsequent pregnancy of each sow. This will ensure that individual differences in antibody response upon basic vaccination of gilts, as observed in our study, will be raised to similar levels during subsequent booster vaccinations. Thus all multiparous sows can be expected to transfer protective antibody levels to their offsprings. Once established, this vaccination program is known to prevent outbreaks of NE in most cases. Combined with good sanitation management, it should keep the environmental contamination with *C. perfringens* type C on the farm low, as newborn piglets should not shed the pathogen in large amounts [5]. Under regular farm management practices in Switzerland, gilts usually represent a maximum of 15–25% of all sows in a breeding farm. Because the infectious pressure and the population of piglets at risk (piglets from gilts) will both be low in properly vaccinated herds, the risk of re-occurrence of the disease can also be considered as low. Nevertheless, under circumstances where the infectious pressure for newborn piglets is high or the population of non-immune and thus highly susceptible piglets is large, immunity of piglets from gilts can easily be increased to achieve a higher level of protection against NE. Examples of such circumstances include cases of acute outbreaks of NE in previously non-vaccinated herds, a management which includes high stocking rates with gilts, or cases of herds, which experience recurrent outbreaks of NE despite vaccination programs.

## Conclusion

We show that currently recommended *C. perfringens* type C vaccination programs induce good levels of antibodies that neutralize the essential virulence factor of *C. perfringens* type C in colostrum and milk as well as piglet serum of multiparous sows. The standard vaccination scheme however might leave a proportion of piglets from gilts susceptible to disease. In cases of recurrent problems, where the immune protection of this subpopulation of piglets in a herd has to be improved, a simple extension of the vaccination scheme for gilts including a basic immunization before insemination and a booster immunization before the first farrowing can be applied to increase neutralizing anti-CPB antibody levels and thus most likely reduce the risk of outbreaks of NE in pig breeding farms.

## Material and methods

Animal experiments were limited to intramuscular (i.m.) injections of licensed and commercially available vaccines or sterile NaCl, blood and colostrum sampling of sows and piglets. They were approved by the Bernese Cantonal Veterinary Office (Animal Experiment No. BE61/16).

### First field trial

Four farms participated in this trial. Three farms had established a continuous vaccination program against *C. perfringens* type C for a minimum of two years. Two farms vaccinated their sows using Porcilis® ColiClos ad us. vet. (MSD Animal Health GmbH; farm A and B) and one farm vaccinated their sows using Clostricol ad us. vet. (Provet AG; farm C). Farm A and B vaccinated their gilts 7 and 3 weeks prior to farrowing and their multiparous sows 3 weeks prior to every consecutive farrowing. Farm C vaccinated their gilts 5 and 2 weeks prior to farrowing and their multiparous sows 2 weeks prior to every consecutive farrowing. The fourth farm served as a control farm, which did not vaccinate their sows against *C. perfringens* type C (farm D).

All participating farms had confirmed absence of clinical and pathological signs of NE for a minimum period of one year prior to the study. On the vaccinating farms, blood and colostrum samples of 9 gilts, 36 multiparous sows (in their 2nd to 9th pregnancy) and 2 piglets per litter were collected. On the non-vaccinating farm, blood and colostrum samples of 2 gilts and 14 multiparous sows as well as 2 piglets per litter were collected. Blood samples were collected 3 weeks before the expected date of delivery (before second immunization in gilts and booster immunization in multiparous sows; T1) as well as in the first 24 h postpartum (*p.p.)* (T2). Colostrum (Col) and milk (M) samples were collected within 24 h (Col) and 2 to 3 days *p.p.* (M). Sera from two piglets per litter were collected 2 days *p.p.* (P).

### Second field trial

The farm with the highest production rate and the most compliant management (farm C) was chosen to test an adapted vaccination scheme using the two commercially available vaccine against *C. perfringens* type C in Switzerland at the time of the start of this trial (October 2017). Sixty-one 6 months old sows were randomly assigned to three groups of 15 and one group of 16 sows respectively. Eight sows developed health issues independent of the vaccination and were excluded from the study.

Group 1.1 and 1.2 were vaccinated using vaccine 1: Porcilis® ColiClos ad us. vet. Vaccine (MSD Animal Health GmbH, Lucerne, Switzerland). Group 1.1 was vaccinated following the manufacturer recommendation against *C. perfringens* type C, which included two injections of 2 ml of the vaccine i.m. 6 (T1) and 2 (T2) weeks *ante-partum* (a.p.). Group 1.2 was vaccinated three times prior to the first farrowing. They received 2 ml of the vaccine i.m. 4 weeks prior to insemination (T0) and at the day of insemination (basic immunization). They received an additional booster vaccination with 2 ml of the vaccine 2 weeks before farrowing. To exclude effects related to the different injection schedules sows in one group received i.m. NaCl injections of 2 ml at those time point when sows from the other groups received their vaccination.

Thus, in addition, gilts from group 1.1 received i.m. injections of 2 ml NaCl 4 weeks prior to insemination (T0) and at the day of insemination. Group 1.2 received i.m. injections of 2 ml NaCl 6 weeks *ante-partum* (T1).

Group 2.1 and 2.2 were vaccinated using vaccine 2: Suisen ad us. vet. Vaccine (Dr. E. Graeub AG, Bern, Switzerland). Again, group 2.1 was vaccinated according to the manufacturer recommendations, twice before the first farrowing. They received 2 ml of the vaccine i.m. 6 (T1) and 3 (T2) weeks a.p.. In addition, they received i.m. injections of 2 ml NaCl 3 weeks prior to insemination (T0) and at day of insemination. Group 2.2 was vaccinated three times prior to the first farrowing. They received 2 ml of the vaccine i.m. 3 weeks prior to insemination (T0) and at day of insemination (basic immunization). They received an additional booster vaccination with 2 ml of the vaccine 3 weeks before farrowing. Group 2.2 received i.m. injections of 2 ml NaCl 6 weeks *ante-partum* (T1).

Blood samples from all gilts were collected prior to insemination (T0), at day of insemination and twice before farrowing (T1 and T2). Colostrum and blood from sows were collected in the first 24 h *p.p.* (Col and T3). Two to three days *p.p.* blood was drawn from two piglets per litter (P). Serum and skimmed milk were generated by centrifugation of blood samples (3,500 x g, 10 min) and colostrum (23,000 x g, 20 min). The samples were stored in aliquots at − 20 °C until examination.

### Determination of neutralizing anti-CPB antibody titers

The cell culture essay using primary porcine aortic endothelial cells (PAEC) was performed as recently described [9]. Briefly, purified recombinant CPB was pre-incubated with serum and colostrum samples of sows and piglets for 1 h at room temperature and then added to PAEC grown in 96-wells at a concentration of 100 ng toxin/ml medium. Cells were incubated at 37 °C for 24 h and subsequently cell vitality was measured using light microscopy and a cell titer blue assay (CellTiter-Blue® Cell Viability Assay, Promega Corporation, Madison, USA). The World Health Organization International Standard Serum of equine origin (antibody titer 4770 IU/mL, National Institute for Biological Standards and Control, Potters Bar, Hertfordshire, EN6 3QG, UK) was used as a positive control and as reference for antibody titer values. Serum derived from non-vaccinated pigs was used as a negative control.

### Statistics

Statistics were performed using the NCSS software (Nashville, USA, http://www.ncss.com). Shapiro–Wilk test was applied to test normality. The logarithm transformation (log(y + 0.01)) was used, as the antibody titers were not normally distributed. The differences in antibody titers between the treatment groups were evaluated by means of a repeated measures analysis of variance, using the animal identification number as a subject variable. The post hoc test applied for multiple comparisons was the Tukey–Kramer Multiple-Comparison Test. The level of significance was defined as 0.05 and 0.01 respectively. The associations between the antibody titers in serum and colostrum was calculated by means of linear regression of the log transformed variables and Spearman-Rank correlation coefficients.

## Data Availability

The datasets used and/or analysed during the current study are available from the corresponding author on reasonable request.

## Abbreviations

a.i.: ante insemination
a.p.: antepartum
*Cl. perfringens*: *Clostridium perfringens*
i.m.: intramuscular
NE: Necrotizing enteritis
p.p.: postpartum
